# Healthcare experiences of gender diverse Australians: a mixed-methods, self-report survey

**DOI:** 10.1186/1471-2458-14-230

**Published:** 2014-03-06

**Authors:** Damien W Riggs, Katrina Coleman, Clemence Due

**Affiliations:** 1 School of Social and Policy Studies, Flinders University, GPO Box 2100, Adelaide 5001, Australia; 2 School of Psychology, The University of Adelaide, Adelaide 5001, Australia

**Keywords:** Gender diversity, Healthcare experiences, Psychiatry, Surgery, Medical education

## Abstract

**Background:**

To date the healthcare experiences of gender diverse Australians have received little attention. Previous international research indicates a range of both negative and positive healthcare experiences amongst this diverse population, with negative experiences being those most frequently reported.

**Method:**

An online survey was designed to examine the healthcare experiences of gender diverse Australians. The survey included Likert scales asking participants to rate their mental and physical health, and their experiences with psychiatrists, general practitioners and surgeons (in terms of perceived comfort, discrimination and information provision). Open-ended questions provided the opportunity for participants to further elaborate on their experiences. Data were collected between June 2012 and July 2013. Quantitative data analysis was conducted utilising SPSS 17.0, including ANCOVAs and correlations to examine the relationships between variables. Qualitative data were coded by the authors in terms of negative or positive responses and the validity of ratings were assessed utilising Cohen's kappa.

**Results:**

110 people assigned male at birth (MAAB) and 78 people assigned female at birth (FAAB) completed two separate surveys. All identified as gender diverse as defined in this paper. 70% of participants had accessed a psychiatrist. Participants MAAB rated their experiences with psychiatrists more highly than participants FAAB. 80% of participants had accessed a general practitioner. Comfort with, and respect from, general practitioners were both positively correlated with mental health, whilst discrimination was negatively correlated with mental health. 42.5% of participants had undertaken sex-affirming surgery. Those who had such surgery reported higher levels of physical and mental health than those who had not undertaken surgery. Participants MAAB reported more positive experiences of surgery than did participants FAAB.

**Conclusions:**

Findings highlight the need for increased education of medical practitioners in regards to engaging with gender diverse clients.

## Background

The role of medical practitioners providing services to gender diverse Australians has increasingly come under scrutiny by practitioners, researchers and community advocates. In western countries such as Australia, gender identity is presumed to be a corollary of assigned sex, with sex assignation determined primarily by visual inspection of the genitalia. This model of sex assignation has historically operated to justify surgeries performed on intersex infants to assign them to one of two sexes, and has also served to perpetuate the assumption that gender identity will accord with assigned sex (where a penis is taken as indicating a male and a vagina is taken as indicating a female). The term ‘gender diverse’ is used in the present paper to refer to people for who this assumption is incorrect. This term does not necessarily encompass the experiences of people born intersex, who were not a focus of the research presented in this paper.

Gender diverse people may enter the healthcare system seeking to access hormone therapies or surgeries aimed at modifying their bodies so that they more closely align with their preferred gender identity. At present, accessing such treatments requires a diagnosis of gender dysphoria, which emphasises the “distress that may accompany the incongruence between one’s experienced or expressed gender and one’s assigned gender” [1]. Practitioners (primarily psychiatrists) are thus charged with the role of assessing for gender dysphoria and prescribing hormone therapies and recommending surgery, if these are what the client desires. Understandably, then, medical practitioners currently have a vital role to play in supporting the wellbeing of gender diverse people.

Research suggests that gender diverse people experience both positive and negative interactions with medical practitioners. In terms of positive experiences, interview research undertaken by Speer and McPhillips with 21 transsexual patients at the UK’s largest gender clinic found that participants classified good psychiatrists as ‘approachable’, ‘easy to talk to’, ‘chatty’ and as having a ‘relaxed interactional style’ [2]. Similarly, Sperber, Landers and Lawrence’s focus group research with 34 transgender people living in Boston found that positive interactions with healthcare professionals were the result of participants feeling that their experiences and needs were heard [3]. Australian research by Pitts and colleagues with 229 gender diverse people suggests that the use of both correct pronouns and clients’ preferred names were conducive to positive interactions [4].

Such positive experiences, however, appear to be the minority in previous research. More common are reports of gender diverse people; 1) being denied services, 2) accessing practitioners with little or no knowledge of the issues facing gender diverse people, 3) being subjected to inappropriate terminology by health professionals, 4) having their own knowledge discounted, and 5) experiencing considerable waiting periods to access services. In terms of the first point, Kenagy’s research with 81 transgender people living in Philadelphia reports that one in four participants had been refused treatment in regards to their gender identity [5]. Sperber, Landers and Lawrence report that one of their participants was refused service, being told to “see a veterinarian instead” [3]. In regards to the second point, empirical research has repeatedly indicated that gender diverse people perceive a lack of knowledge on the part of medical practitioners, and that they therefore experience an injunction to educate practitioners in order to receive services. For example, Grant and colleagues report that 50% of participants in a sample of 6,450 transgender people stated that they had to educate their health care provider on transgender health issues [6]. Xavier, Hannold, Bradford and Simmons similarly suggest that 46.3% of their sample of 350 transgender individuals reported the need to educate their providers [7]. This lack of knowledge is reflected in the common reporting of medical practitioners using inappropriate terminology when engaging with gender diverse clients. For example, participants in two previous studies indicated that they were frequently misgendered by medical practitioners (i.e., they were referred to by their natally assigned sex rather than by their current gender identity), with some participants even reporting being referred to as ‘it’ [3,8]. Notably, however, whilst the need to educate healthcare professionals appears common, previous research has also emphasised that practitioners often discount the knowledge held by gender diverse clients. For example, Poteat, German and Kerrigan’s interview research with 55 transgender people living in a small mid-Atlantic city indicates that some participants were told that they had “read too much on the internet” [9]. Finally, a survey of 889 transgender people living in the UK indicates that 32% of the sample waited between one and three years to access services, and 10% waited more than three years. 58% of the sample indicated that such lengthy waiting periods had negatively contributed to poor mental health outcomes [10].

These extensive findings of negative experiences with healthcare providers suggest that at present a significant majority of gender diverse people receive inadequate service. This is of concern given the fact that some gender diverse people who fear or have negative interactions with medical practitioners may seek to access hormones online, a practice that brings with it considerable risks [11]. Given the fact that gender diverse people are at risk of higher rates of mental health concerns and suicidality arising from living in discriminatory social contexts [6,12]., feeling unable to access healthcare services may further exacerbate these risks. The research reported in this paper sought to further examine the experiences of gender diverse Australians, and to identify the relationships between a number of demographic and health variables and healthcare experiences.

## Method

Participants were 188 people who completed one of two surveys conducted over two successive six-month periods aimed at ascertaining the health care experiences of people whose gender identity would be classified as ‘gender diverse’ as defined above. One survey, completed by 110 people who were male assigned at birth (MAAB) but who do not identify as male, utilised the term ‘transgender women’ in the information provided to potential participants. The second survey, completed by 78 people who were female assigned at birth (FAAB) but who do not identify as female, utilised the term FAAB in the information provided to potential participants. In response to a question asking people to describe their gender identity, terms such as ‘man’, ‘woman’, ‘trans man’, ‘trans woman’, and ‘genderqueer’ were used. Statistical testing indicated no significant differences between these terms in regards to responses to the survey items, hence the term ‘gender diverse’ is used in the present paper to describe all participants as a group. The key point of statistical difference was in terms of assigned sex, and thus this distinction is retained for the purposes of analysis, with the terms MAAB and FAAB utilised where relevant. The mean age for participants was 44.87 years (SD = 13.93). Participants MAAB were older (M = 44.94, SD = 13.92) than participants FAAB (M = 32.05, SD = 10.71), t(183) = 6.84, p < .001. In terms of cultural identity, almost all respondents (94%) identified in some way as white Australians. People used terms such as ‘white’, ‘Anglo’, and ‘Anglo- European’. 3% of all respondents identified as Indigenous. 2% of all respondents identified as Asian. 1% of all respondents identified as Middle Eastern.

Participants completed one of two online surveys administered via SurveyMonkey (the only differences being the language used in the description for each survey). Items of relevance to the present paper asked participants to rate their experiences of comfort with, and respect from, general practitioners on a Likert scale where 1 = very negative, 2 = negative, 3 = neutral, 4 = positive, and 5 = Very positive. This same scale was used to ask about experiences with psychiatrists, experiences of surgery, and experiences of post-surgery care. Participants also rated whether or not they had experienced discrimination in their interactions with general practitioners, and whether or not they felt they had to educate their general practitioner, on a Likert scale where 1 = Not at all, 2 = Somewhat, 3 = Neither yes nor no, 4 = Often, and 5 = Always. This same scale was used to ask about information provision prior to surgery, and degree of perceived control over surgery. Participants rated their physical and mental health on a Likert scale where 1 = Very bad, 2 = Bad, 3 = Neutral, 4 = Good, and 5 = Very good. Participants were also asked to provide comments about their experiences with medical practitioners via open-ended responses, with questions including “Could you please described your experiences with general practitioners/psychiatrists/surgeons”.

Quantitative data were analysed utilising SPSS 17.0. Open-ended responses were independently coded by each of the authors on the basis of whether they included primarily negative accounts of experiences with medical professionals or primarily positive accounts of healthcare professionals. Cohen’s kappa for assessing inter-rater reliability was calculated for both positive and negative responses. Kappa scores for the rating of each category were high, ϰ = 0.92, *p* < .0.001 (positive) and ϰ = 0.89, *p* < .0.001 (negative). Any differences in evaluation were resolved by discussion between the three authors. Due to the fact that many of the open-ended responses contained personal identifying information in regards to either participants or medical professionals, only a limited number of indicative examples are given below. Prevalence rates are provided for negative and positive responses to open-ended questions, and chi square tests are reported in regards to responses differentiated by assigned sex where relevant. It should be noted that not all participants provided open-ended responses.

Ethics approval was granted by the Flinders University Social and Behavioural Research Ethics Committee, approval number 5656. Participants were sourced using snowball sampling methods, and no direct approaches were made to potential participants by the researchers. All participants gave their informed consent to participate in the study by reading an information screen at the beginning of the online survey, and then either choosing to continue (by giving their consent to be involved and have their data included in the research), or decline by simply choosing not to proceed to the survey. Information provided to participants assured their anonymity and indicated that findings would be reported in peer-reviewed publications.

## Results

### Experiences with psychiatrists

70% of participants had accessed a psychiatrist. Participants MAAB on average rated their experience as just below ‘positive’ (M = 3.65, SD = 1.25). People FAAB on average rated their experience as just below neutral (M = 2.73, SD = 1.25). To determine if these differences were significant, and given the age differences between the two cohorts, an ANCOVA was performed with satisfaction with psychiatrists as the dependent variable, assigned sex as the fixed factor, and age as the covariate. There was a significant effect of assigned sex on satisfaction with psychiatrists even when controlling for age, F(1,115) = 4.479, p < .05.

Analysis of open-ended responses (n = 110) suggested that positive experiences (n = 43) were marked by psychiatrists being, for example, “professional, helpful, knowledgeable” and being “a caring, humane practitioner”. Negative experiences (n = 67) were marked by a concern that seeing a psychiatrist was, for example, “a gatekeeping exercise”, or involved being “asked lots of ridiculous or offensive questions”. Participants MAAB were more likely to report positive experiences than were participants FAAB, *X*^2^(1, N = 43) = 9.72, p < .01. Participants FAAB were more likely to report negative experiences than were participants MAAB, *X*^2^(1, N = 67) = 8.64, p < .01.

### Experiences with general practitioners

80% of participants had accessed a general practitioner. Participants on average were positive about feeling respected by general practitioners (M = 4.19, SD = 1.18), though some discrimination was reported (M = 1.70, SD = 1.22). Participants on average were neutral about the degree of comfort they felt with general practitioners (M = 3.61, SD = 1.53). There were no significant differences between ratings in terms of assigned sex.

There was a moderate positive relationship between feeling greater comfort with General Practitioners (GPs) and levels of self-reported mental wellbeing, r = .39, p < .001, and also a moderate positive relationship between feeling respect from GPs, and levels of self-reported positive mental wellbeing r = .41, p < .001. Conversely, there was a negative relationship between reported levels of discrimination from General Practitioners and levels of self-reported mental wellbeing, r = −.42, p < .001. Finally, there was a moderate positive relationship between the need to educate GPs and levels of discrimination, r = .31, p < .001, and a moderate negative relationship between the need to education GPs and feeling respected, r = −.38, p < .001.

Analysis of open-ended responses (n = 147) suggested that positive experiences (n = 85) primarily emphasised, for example, “being treated with respect”, whilst negative experiences (n = 62) involved feeling that physical healthcare can, for example, “be invasive and sometimes abusive” and that there was often a need “to educate them”. There were no significant differences between positive and negative responses in terms of assigned sex.

### Experiences of surgery

Overall, 42.5% of participants had undertaken sex-affirming surgery. On average participants reported positive experiences with surgery (M = 4.27, SD = 0.89), as well as positive experiences of post surgery support (M = 4.34, SD = 0.68). On average participants felt that their surgeon had provided adequate information about the surgery (M = 4.64, SD = 0.73), and that they felt they had control over the surgery process (M = 4.49, SD = 0.74).

Participants MAAB who had surgery reported more positive experiences of surgery (M = 4.27, SD = 0.89) than did participants FAAB (M = 1.66, SD = 1.09), t(74) = 11.42, p < .001. Participants MAAB who had surgery also reported more positive experiences of post-surgery care (M = 4.34, SD = 0.68) than did participants FAAB (M = 1.84, SD = 1.16), t(74) = 11.73, p < .001. Participants MAAB were more likely to have undertaken surgery than were participants FAAB, X2 = 4.54, p < .05.

An ANCOVA was performed to assess the relationship between surgery and mental health, with age as the covariate. Having had surgery (M = 3.87, SD = 0.98) or not (M = 2.98, SD = 1.16) was a significant predictor of mental health, F(1, 167) = 3.738, p < .05. An ANCOVA with age as the covariate was also performed with physical health as the dependant variable. Those who had surgery reported higher levels of self-reported physical health (M = 4.00, SD = 0.90) than did those who had not undertaken surgery (M = 3.10, SD = .98), F(1, 167) = 4.531, p < .01.

Analysis of open-ended responses (n = 65) suggested that positive experiences (n = 40) were marked by surgeons being, for example, “willing to work with me to achieve my desired outcome”. Negative experiences (n = 25) of surgery involved occurrences such as “I was not adequately warned of the possible scarring”, and “some staff have shared my information without permission and it can be embarrassing”. Participants MAAB were more likely to report positive experiences than were participants FAAB, *X*^2^(1, N = 40) = 10.42, p < .01. Participants FAAB were more likely to report negative experiences than were participants MAAB, *X*^2^(1, N = 25) = 11.52, p < .01.

## Discussion

These findings clearly indicate both that some medical professionals are doing well in servicing the healthcare needs of gender diverse clients in Australia, whilst other professionals are falling short of adequately meeting these needs. The findings echo previous research, highlighting experiences of misgendering, the need to educate professionals, and discriminatory comments being made. It is notable that for both psychiatrists and surgeons, participants MAAB had more positive experiences than did participants FAAB. This may be indicative of the fact that surgeries related to sex characteristics for people MAAB in Australia (i.e., vaginoplasty) are more widely available than are surgeries for people FAAB (i.e., phalloplasty). Given the fact that the sample included greater numbers of people MAAB, and that a significant number of these participants had undertaken surgery, this may account for the more positive depiction of medical practitioners reported across the sample as a whole. In other words, if both general practitioners and psychiatrists have a significant gatekeeping role to play in accessing hormone therapies and surgeries for gender diverse people in Australia, and if a significant number of participants were able to access medical practitioners willing to support their wish for hormone therapies and surgeries, then this logically would account for the primarily positive experiences reported by participants. That a significant number of participants FAAB still reported being less satisfied with their experienced than participants MAAB potentially indicates the differential effects both of gatekeeping practices and the availability of surgery for people FAAB.

A key limitation of the research was the reliance upon single item measures for all variables. Future research will benefit from the increased use of composite scales to assess health and wellbeing measures, as well as measures of comfort. Future research may also focus upon the apparent distinction between participants on the basis of assigned sex. Whether this is a product of the sample or a product of differences between how medical professionals engage with gender diverse people on the basis of their assigned sex requires further examination.

## Conclusions

Limitations aside, the findings indicate the need for better education of medical professionals in regards to engaging with gender diverse clients. That such clients might be at risk of self-harm or poor mental health as a result of negative interactions with medical professionals suggests that considerable priority must be accorded to the provision of such education. Whilst it is likely to remain the case that medical professionals will continue to play something of a gatekeeping role - particularly with regard to accessing Medicare funded therapies and treatments - more supportive and inclusive approaches to practice may help to ameliorate gender diverse clients’ feelings of discrimination and discomfort.

As the findings indicate, this may be especially so for people FAAB, who appeared to have less positive experiences with medical professionals. This would suggest the pressing need for more training of Australian surgeons to increase the availability of surgery (e.g., phalloplasty) for people FAAB in Australia (such surgeries are currently primarily only available outside of Australia). This may help to increase satisfaction amongst clients who were FAAB (and who desire such surgeries). Looking more broadly beyond a narrow focus on surgery, it would appear evident that service provision to gender diverse Australians would be greatly improved by mandatory training for all medical professionals so as to widen the range of options that clients have in selecting a medical professional who they feel can best meet their needs. Increasing the number of medical professionals capable of providing competent care to gender diverse Australians will likely make a significant contribution to the overall wellbeing of this population.

## Competing interests

The authors declare that they have no competing interests.

## Authors’ contributions

DR conceived of the study and its initial design. DR and CD then designed and wrote the survey questions, with DR and KC recruiting participants. DR and KC carried out initial analysis, with CD checking these. KC drafted the initial manuscript, with comments and suggestions made by DR and CD. All authors read and approved the final manuscript.

## Pre-publication history

The pre-publication history for this paper can be accessed here:

http://www.biomedcentral.com/1471-2458/14/230/prepub
